# Estimation of methane gas by LandGEM model from Yasuj municipal solid waste landfill, Iran

**DOI:** 10.1016/j.mex.2019.02.013

**Published:** 2019-02-15

**Authors:** Saeid Fallahizadeh, Masoumeh Rahmatinia, Zakarya Mohammadi, Marzieh Vaezzadeh, Ali Tajamiri, Hamed Soleimani

**Affiliations:** a Research Center for Environmental Health Technology, Iran University of Medical Sciences, Iran; b Department of Environmental Health Engineering, School of Public Health, Iran University of Medical Sciences, Tehran, Iran; c Student Research Committee, Department of Environmental Health Engineering, School of Public Health and Safety, Shahid Beheshti University of Medical Sciences, Tehran, Iran; d Department of Environmental Health Engineering, Faculty of Health, Hormozgan University of Medical Sciences, Bandar Abbas, Iran; e Department of Environmental Health, School of Health, Guilan University of Medical Sciences, Rasht, Iran; f Municipality of Yasuj, Urban Services Office, Yasuj, Iran; g Department of Environmental Health Engineering, School of Public Health, Tehran University of Medical Sciences, Tehran, Iran

**Keywords:** LandGEM model, Municipal solid waste, MSW, LandGEM, Methane, Yasuj

## Abstract

CH_4_ has a high potential for energy production and by utilizing the proper technology, large amounts of energy can be extracted from it. This study aimed to estimate the amounts of methane emissions from the municipal solid waste landfill in Yasuj city using LandGEM software. The LandGEM model which is used for this aim is based on input data of open landfill year, land closure year, methane production rate, potential methane production capacity, and waste acceptance rate. According to the results, methane gas production during the years 2009, 2010, 2011 and 2012 was obtained to be 250, 275, 303, and 330 m^3^/h respectively. The results also showed that maximum methane production rate occurred during the years 2010–2012 and then reduced with a soft slope from 2012. The method and results of this research can be used for design and execute of methane gas collection systems and also, control of greenhouse gases emission for the landfills.

## Highlights

• Emissions of methane and carbon dioxide gases from Yasuj landfill were estimated by LandGEM software.
• The results indicated that methane could be used as a suitable energy source.
• The results also indicated that proper burial of MSW can be useful for controlling emissions of the greenhouse gases.

## Graphical abstract

**Figure:**
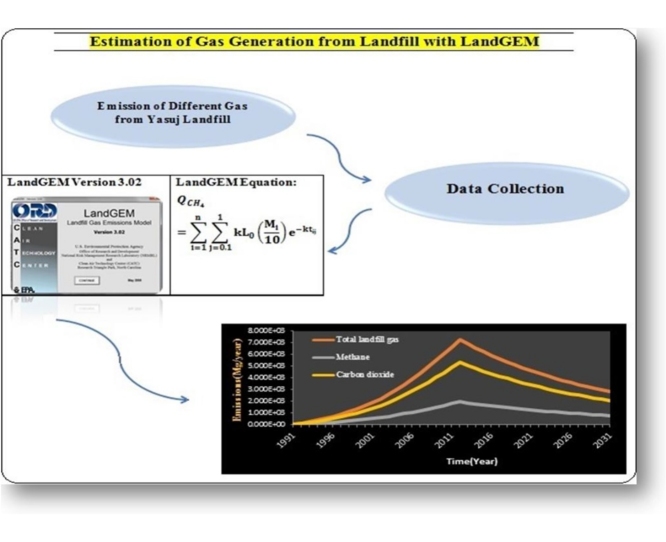

**Specifications Table**

**Table:** 

Subject area	Environmental Sciences. Solid waste management
More specific subject area	Landfill
Method name	LandGEM model
Name and reference of the original method	Talaiekhozani A, Bahrami S, Hashemi SMJ, Jorfi S. Evaluation and analysis of gaseous emission in landfill area and estimation of its pollutants dispersion, (case of Rodan in Hormozgan, Iran). Environmental Health Engineering and Management Journal. 2016;3(3):143-50. [1]
Resource availability	Data is presented in this article.

## Introduction

The production of municipal solid waste (MSW) increases with socio-economic condition development [2,3]. The climate, political and cultural conditions of Yasuj city and the growing development of industries and services have led to rapid population growth so that the city's population growth rate has been about 73.8 percent over the past 20 years (1991–2010). Rapid population growth has further added to the amount of municipal solid waste production. Recently, the government of Iran has a policy that each province or city should have a comprehensive solid waste management plan. However, many technical and economic problems prevent a comprehensive waste management plan. As landfill is a low-cost and technically feasible method, has a high of attention in solid waste management plans [2,4,5]. The unavoidable after-effects of solid waste disposal in a landfill are gas and leachate generation due to climate condition, microbial disintegration, refuse properties and landfilling operations [6,7].

Landfill gas (LFG) is produced from the biological and chemical processes that occur when waste buries in the landfill. The primary components of LFG are carbon dioxide (40–50%) and methane (50–60%) [[8], [9], [10], [11]]. Landfill gas is produced by microbial activity on biodegradable wastes under anaerobic conditions [12]. Methane and carbon dioxide are the significant components of landfill gas and actively contribute to the greenhouse effect [13]. Also, methane gas is one of the most important greenhouse gases that has a potential of 21 times more than carbon dioxide gas in global warming [14]. However, in the most cases, the waste will be covered by 10 to 15 cm of the soil to reduce the gas and odor emission, but the little amount of these gases continues to release into the atmosphere [15,16]. Many studies have been carried out to estimate the amount of gas production from municipal waste landfills. So, different models have been designed to calculate greenhouse gas emissions, oxidation, and also landfill gas production. In the case of modeling the landfill process, the importance of local factors, such as the waste composition, disposal, and protection systems against potential impacts, leads to the development of models used for various landfill facilities [17]. Modeling and predicting the production rate of methane gas in the landfill is very significant for designing and operating sites such as Yasuj landfill. Measuring the amount of methane gas emissions from landfills could help in determining Iran's contribution to global emissions of greenhouse gases (GHGs).

There are several methods of evaluating the methane emissions, including field experiment, site appraisement, and mathematical modeling [18,19]. In a study by Sadeghi et al. [20], the results showed that during 2018, 2023, 2028 and 2033 gas production is supposed to be 205, 410, 549 and 671 m3/h, respectively [20]. Also, many researches have been carried out about the estimation of methane gas production from landfils[21,22]. Yasuj city is located in the south of Iran, has a landfill receiving than 120 tons of waste every day. There are no facilities for methane recovery in this area. Due to lack of information about the evaluating methane gas emission from Yasuj municipal solid waste landfill, this research aimed to describe the Land GEM model for gas emission in Yasuj municipal solid waste landfill with the estimation results of carbon dioxide and methane production.

## Materials and methods

### Study area

This research is a descriptive-cross sectional study in which Land GEM software was used to estimate the methane rate produced at Yasuj landfill. Yasuj landfill site with an area of about 25 ha was started in 1991, at a distance of 26 km from the city center and in Yasuj-Babameydan road in a area called dupshteh dashtrom. The trench method is used for waste burial in landfill site including excavated channels at depths of 5 m, 4 m’ width and 50 m in length. Then the buried waste is covered with a layer of soil at a depth of 15 to 30 cm. Also, the soil type of landfill site is a sandy-clay.

### Data collection

First, the data related to Yasuj landfill and demographic data were collected based on population annual growth rate over different years. Then, the required data including potential of methane production capacity, constant methane value, and content (% by volume) were entered into the software and finally the methane emissions were computed. According to the general census in 1991, the population growth rate in Yasuj was 10.8%, and in 2010, thisrate reached 1.78%. Yasuj is highly migratory due to its climatic and geographical, political and cultural situation. Based on comprehensive waste management plan in Yasuj, the period of the selected plan for Yasuj landfill was 20 years. In order to calculate the amount of methane emissions by LandGEM software, the weight of wastes produced during plan period must be suitably evaluated. LandGEM determines the methane mass produced by using the mass of waste deposited and the methane generation capacity.

### The existing situation of the area

Based on study conducted in 2010 in Yasuj, waste generation per capita was 1028 g/d and in the surrounding villages was 408 g/d [23]. The average density measured in Yasuj is 365 kg/m^3^ for residential areas and 470 kg/m^3^ for residential-commercial areas, as well as for the amount of moisture content of spoilable waste in the range of 30 to 50 percentage [23]. As shown in Table 1, based on the results regarding the components of MSW in Yasuj, the highest amount of waste is related to food waste and is next to plastic, paper and cardboard, glass, and textiles. The information on characteristics of waste generated and disposed of in landfill of Yasuj for 1991–2010 years adopted from the study of Mirbagheri et al. [23].

**Table 1:** Components of Yasuj municipal solid waste [23].

Waste components	Weight average (%)	Waste components	Weight average (%)
Food waste	76.6	Textiles	1.4
Paper and cardboard	4.8	Glass	2.4
Diaper	3.8	Iron metals	1.1
Plastics	6.4	Non-iron metals	0.33
PET	1.5	Bread waste	0.63
Rubber and leather	0.2	Others	0.84

### Description of the LandGEM model

The LandGEM software was extended by US EPA’s specialists to fetch most of the enormous US landfills into the air quality surveillance program (under Clean Air Act amendments) and to extend them for local emission stocks. Increasing from first-order to second-order makes the modeling manner more wrapped and is not explained with increasing accuracy [24]. Therefore most models are used based on a first-order equation, such as shown in below equation. Two important parameters used in modeling LFG generation based on a first-order equation are the methane production rate constant, k (yr^−1^) and the methane production potential, L_0_ (m^3^/ Mg).

$$Q_{{CH}_{4}}=\sum_{\text{i}=1}^{\text{n}}\sum_{\text{j}=0.1}^{1}\text{k}{\text{L}}_{0}\left(\frac{{\text{M}}_{\text{i}}}{10}\right){\text{e}}^{-\text{k}{\text{t}}_{\text{i}\text{j}}}$$

Where Q_CH4_ is the amount of annual methane generation in the year (m_CH4_^3^/year); i is the one-year time increment; n defines as (year of the calculation) - (initial year of waste acceptance). Also, *j* is 0.1 year time increment; k is the methane production rate (year^−1^); L_o_ is the potential methane production capacity (m^3^/Mg); M_i_ is the mass of waste accepted in the ith year (Mg); tij is the age of the jth sector of waste mass, Mi accepted in the i_th_ year (decimal years, e.g., 3.2 years). Also in this study, model parameters from user inputs are k = 0.050 Year^−1^, L_o_ = 170 m^3^/Mg, and methane content 50% by volume. It is managed by two main factors, the decay rate (k) and the methane potential capacity (L_0_) of landfill waste [25]. The methane potential of waste depends on biodegradable waste quantity, separation level, microbial usage rates, volatile solids, climatic conditions like humidity and temperature [26,27]. This model has been originated based on climatic conditions and waste specifications of USA [28]. The k determines methane generation rate for the mass of waste in the landfill. The higher value of k, the rate of methane production increases faster and then decomposes over time. The value of k is depended primarily to four factors: 1) Availability of the nutrients for microorganisms that break down the waste to form carbon dioxide and methane, 2) pH of the waste mass, 3) Moisture content of the waste mass and 4) Temperature of the waste mass [29]. To evaluation L_0_, the methane yields of single waste components were pursued from default data of software model and literature regarding wastes generated in the USA [30,31] because of the lack of data on the properties of Yasuj MSW. In this study, according to Table 2 based on LandGEM software data, the default values of k and L_0_ are used 0.05 year^−1^ and 170 m^3^/Mg for Yasuj respectively. Despite the high organic matter content (food waste) a weight percentage of 76.6%, the value of k could have been higher in Yasuj MSW but was considered the same as the default value. Characteristics of model parameters to run the Land GEM adopted from Alexander et al. (2005) (29) that has been indicated in Table 2.

**Table 2:** Determine model parameters to run the LandGEM [29].

Parameters	Reference	Unit	Symbol	Rate
Methane production	CAA^a^	year^−1^	k	0.05
Potential methane production capacity	CAA	m^3^/Mg	L_0_	170
NMOC concentration	CAA	ppmv as hexane	–	4000
Methane content	CAA	by volume	–	50

a Model parameters according to Clean Air (CAA) Regulations.

## Result and discussion

Table 3 shows the result of the disposed solid waste quantity of Yasuj during the 20 years of open landfill years. The amount of disposed of municipal waste produced was approximately estimated 5,200 Mg in 1991 which increased to 43,000 Mg in 2010. According to Table 3 total quantity of disposed waste, 466,000 short tons estimated at this landfill by the year of 2010. These digits show the rapid increase in municipal waste production in this area due to population growth and increasing the industrial and commercial sections. Methane emission estimates based on LandGEM is the practical method in nature which vary according to the landfill management and waste composition were considered while developing this method [15].

**Table 3:** Input data sheet to the LandGEM software for Yasuj.

Year	Waste Accepted		Waste-In-Place		Year	Waste Accepted		Waste-In-Place	
	(Mg/year)	(short tons/year)	(Mg)	(short tons)		(Mg/year)	(short tons/year)	(Mg)	(short tons)
1991	5,233	5756	0	0	2001	19,780	21,758	105,795	116,374
1992	5,899	6,489	5,233	5756	2002	23,394	25,734	125,574	138,131
1993	6,871	7,558	11,131	12,245	2003	25,076	27,583	148,968	163,865
1994	8,216	9,038	18,002	19,803	2004	28,769	31,646	174,044	191,449
1995	9,581	10,540	26,218	28,840	2005	31,196	34,316	202,814	223,095
1996	10,887	11,975	35,800	39,380	2006	33,237	36,560	234,010	257,411
1997	12,068	13,275	46,687	51,355	2007	35,671	39,238	267,247	293,971
1998	13,643	15,008	58,755	64,630	2008	37,491	41,240	302,917	333,209
1999	15,798	17,378	72,398	79,638	2009	40,196	44,216	340,408	374,449
2000	17,598	19,358	88,196	97,016	2010	42,973	47,270	380,605	418,665

The level of food culture and tastes in Iran is such that still a significant part of the waste is food waste, and this applies to Yasuj. However, in other countries, this matter is lower and even reduced by half. According to research conducted by kalantarifard et al. the amount of food waste is 30% in Malaysia [15]. However, Table 1 shows waste composition especially food waste contains about 76.6% in the waste stream to this landfill. The half-life of organic matter such as food waste is too short for methane production in Landfill. Already, Tsatsarelis et al. research in 2009 revealed this subject and stated that the production of methane for the half-life of textiles and wood between 15 and 30 years seems to be almost the same, indicating that its changes also do not significantly affect. Therefore, it is expected that the values of wood and textiles are small in comparison to the other materials of disposed of in a landfill, which leads to a relatively small contribution to methane production [32]. Table 4 shows the annual methane production from disposed waste at the landfill, and methane production increased over time. According to Table 4, the total production of methane in 1992 was 2.902 × 10^1^ Mg, which increased to 1.610 × 10^3^ in 2010. In other words, The amount of methane production has risen from 0.5% to 3.75%.

**Table 4:** The number of production gases based on the LANDGEM software for Yasuj.

Year	Total landfill gas	Methane	Carbon dioxide	Year	Total landfill gas	Methane	Carbon dioxide
	(Mg/year)	(Mg/year)	(Mg/year)		(Mg/year)	(Mg/year)	(Mg/year)
1991	0	0	0	2001	1.867E+03	4.988E+02	1.369E+03
1992	1.086E+02	2.902E+01	7.961E+01	2002	2.187E+03	5.842E+02	1.603E+03
1993	2.258E+02	6.031E+01	1.655E+02	2003	2.566E+03	6.854E+02	1.881E+03
1994	3.574E+02	9.547E+01	2.619E+02	2004	2.961E+03	7.910E+02	2.170E+03
1995	5.105E+02	1.364E+02	3.742E+02	2005	3.414E+03	9.120E+02	2.502E+03
1996	6.846E+02	1.829E+02	5.017E+02	2006	3.895E+03	1.040E+03	2.855E+03
1997	8.772E+02	2.343E+02	6.429E+02	2007	4.395E+03	1.174E+03	3.221E+03
1998	1.085E+03	2.898E+02	7.951E+02	2008	4.921E+03	1.315E+03	3.607E+03
1999	1.315E+03	3.513E+02	9.639E+02	2009	5.460E+03	1.458E+03	4.001E+03
2000	1.579E+03	4.218E+02	1.157E+03	2010	6.028E+03	1.610E+03	4.418E+03

**Fig. 1:**
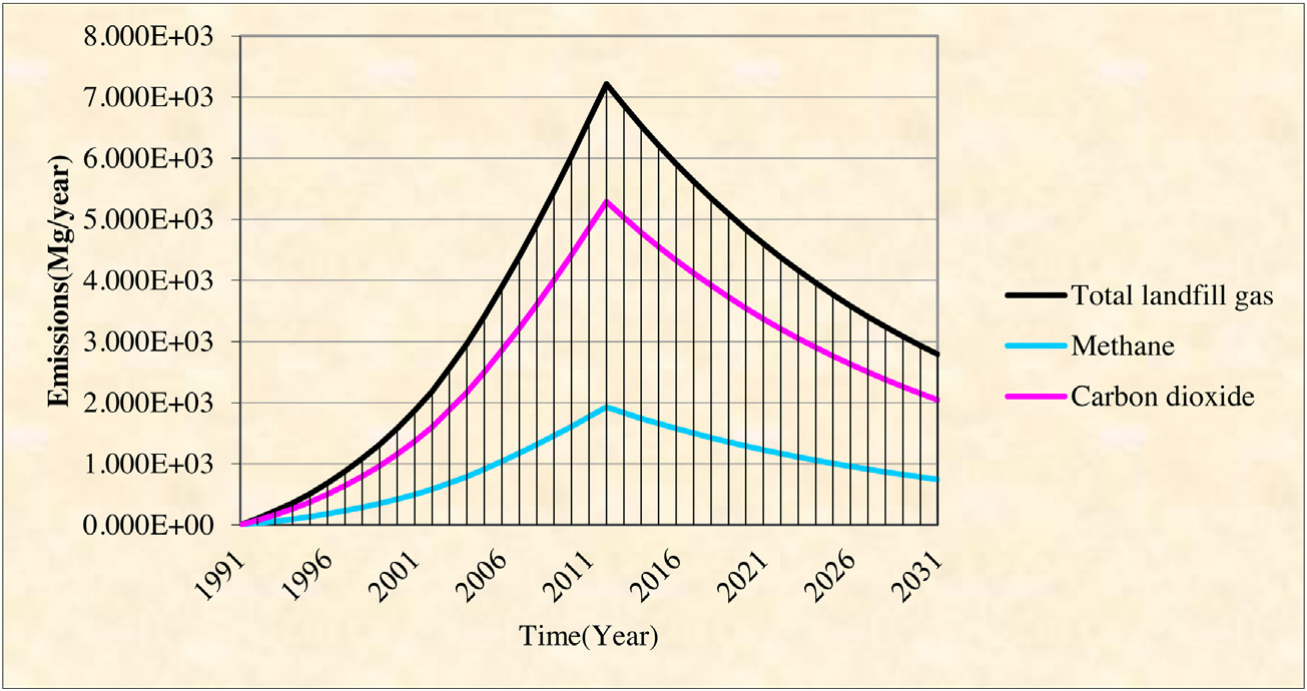
The amount of gas emission from Yasuj landfill site from year 1991 to 2031.

**Fig. 2:**
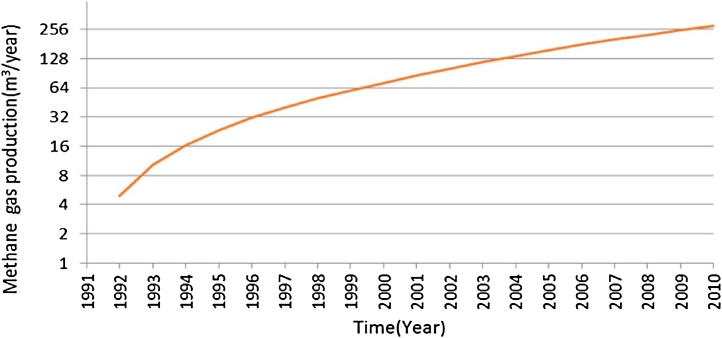
The amount of gas production from Yasuj landfill site from 1991 to 2010.

Fig. 1 shows the trend of methane gas emission in different years of the project at the waste disposal site of Yasuj. The results showed that the amount of annually solid waste production in the landfill of Yasuj was varied from 5756 tons to 42,973 tons from the open landfill to the closure landfill. As Fig. 2 shows, the amount of methane gas production was 5 m^3^/h and 275 m^3^/h in 1992 and 2010 respectively.

Sadeghi et al. study [20] in Sanandaj showed that methane production rate in the first year of landfill operation was 10 m^3^/h and reached 671 m^3^/h over the period of 20 years (2014–2033) [20]. Comparison of this study with the results of Sadeghi et al. showed that the amount of methane production in Yasuj is lower, due to the lower population and the amount of waste produced. On the other hand, the difference in the figure shows that population growth has been 73.8% over the past 20 years.

## Conclusion

The methane gas emission has been estimated using the LandGEM model for Yasuj landfill. This landfill starts operation at 1991 with the objective to receive the produced solid waste at the area until 2010. The amount of methane production from solid waste calculated from Have we correctly interpreted the following funding source(s) and country names you cited in your article:2.902 × 10^1^ (Mg/year) in 1992, the first year after waste acceptance by landfill while the maximum methane production rate occurred during the years 2010–2012 were indicated as the peak of production by 1.928 × 10^3^ (Mg/year). The results of the current research can be used to calculating in the energy production planning and other uses from landfill gas and as well as determining Iran contribution of global emissions of greenhouse gases. Moreover, due to the amount of calculated methane gas, it is possible to design and execute methane gas collection systems for each burial site, in order to prevent the use of gas from its accumulation in burial places and to prevent explosions and possible deposition.

## References

[1] Talaiekhozani A., Bahrami S., Hashemi S.M.J., Jorfi S. (2016). Evaluation and analysis of gaseous emission in landfill area and estimation of its pollutants dispersion, (case of Rodan in Hormozgan, Iran). Environ. Health Eng. Manage. J..

[2] Calabrò P., Gentili E., Meoni C., Orsi S., Komilis D. (2018). Effect of the recirculation of a reverse osmosis concentrate on leachate generation: a case study in an Italian landfill. Waste Manag..

[3] Pirsaheb M., Khosravi T., Sharafi K. (2013). Domestic scale vermicomposting for solid waste management. Int. J. Recycl. Org. Waste Agric..

[4] Pirsaheb M., Azizi E., Almasi A., Soltanian M., Khosravi T., Ghayebzadeh M. (2016). Evaluating the efficiency of electrochemical process in removing COD and NH4-N from landfill leachate. Desalin. Water Treat..

[5] Sharafi K., Pirsaheb M., Maleki S., Arfaeinia H., Karimyan K., Moradi M. (2018). Knowledge, attitude and practices of farmers about pesticide use, risks, and wastes; a cross-sectional study (Kermanshah, Iran). Sci. Total Environ..

[6] Finnveden G., Johansson J., Lind P., Moberg Å. (2005). Life cycle assessment of energy from solid waste—part 1: general methodology and results. J. Clean. Prod..

[7] Moberg Å, Finnveden G., Johansson J., Lind P. (2005). Life cycle assessment of energy from solid waste—part 2: landfilling compared to other treatment methods. J. Clean. Prod..

[8] Amini H.R., Reinhart D.R., Mackie K.R. (2012). Determination of first-order landfill gas modeling parameters and uncertainties. Waste Manag..

[9] Spokas K., Bogner J., Chanton J., Morcet M., Aran C., Graff C. (2006). Methane mass balance at three landfill sites: what is the efficiency of capture by gas collection systems?. Waste Manag..

[10] Aghdam E.F., Scheutz C., Kjeldsen P. (2018). Impact of meteorological parameters on extracted landfill gas composition and flow. Waste Management.

[11] Pasalari H., Farzadkia M., Gholami M., Emamjomeh M.M. (2018). Management of landfill leachate in Iran: valorization, characteristics, and environmental approaches. Environ. Chem. Lett..

[12] Couth R., Trois C., Vaughan-Jones S. (2011). Modelling of greenhouse gas emissions from municipal solid waste disposal in Africa. Int. J. Greenh. Gas Control..

[13] Chiriac R., Carre J., Perrodin Y., Fine L., Letoffe J.-M. (2007). Characterisation of VOCs emitted by open cells receiving municipal solid waste. J. Hazard. Mater..

[14] Nikkhah A., Khojastehpour M., Abbaspour-Fard M.H. (2018). Hybrid landfill gas emissions modeling and life cycle assessment for determining the appropriate period to install biogas system. J. Clean. Prod..

[15] Kalantarifard A., Yang G.S. (2012). Estimation of methane production by LANDGEM simulation model from Tanjung Langsat municipal solid waste landfill, Malaysia. Int. J. Sci. Technol..

[16] Kalantarifard A., Yang G.S. (2011). Energy potential from municipal solid waste in Tanjung Langsat landfill, Johor, Malaysia. Int. J. Eng. Sci. Technol. (IJEST).

[17] Mohareb A.K., Warith M.A., Diaz R. (2008). Modelling greenhouse gas emissions for municipal solid waste management strategies in Ottawa, Ontario, Canada. Resour. Conserv. Recycl..

[18] Chiemchaisri C., Visvanathan C. (2008). Greenhouse gas emission potential of the municipal solid waste disposal sites in Thailand. J. Air Waste Manage. Assoc..

[19] Di Bella G., Di Trapani D., Viviani G. (2011). Evaluation of methane emissions from Palermo municipal landfill: comparison between field measurements and models. Waste Manag..

[20] Sadeghi S., Shahmoradi B., Maleki A. (2015). Estimating methane gas generation rate from Sanandaj City Landfill using LANDGEM software. Res. J. Environ. Sci..

[21] Hosseini S., Yaghmaeian K., Yousefi N., Mahvi A. (2018). Estimation of landfill gas generation in a municipal solid waste disposal site by LandGEM mathematical model. Glob. J. Environ. Sci. and Manage..

[22] Biglari H., Rahdar S., Baneshi M.M., Ahamadabadi M., Saeidi M., Narooie M.R. (2017). Estimating the amount of methane gas generated from the solid waste using the land GEM software, sistan and baluchistan. J. Glob. Pharma Technol..

[23] Mirbagheri S.A., Jamshidi A., Tajamiri A. (2013). An Investigation of Yasuj Landfill Leachate and its Impact on Water Quality Parameters of Shahqasem Dam and Tangkonareh Wells Water Resources. Islamic azad university.

[24] Oonk H., Boom T. (1995). Validation of landfill gas formation models. Stud. Environ. Sci..

[25] Cho H.S., Moon H.S., Kim J.Y. (2012). Effect of quantity and composition of waste on the prediction of annual methane potential from landfills. Bioresour. Technol..

[26] Xiaoli C., Ziyang L., Shimaoka T., Nakayama H., Ying Z., Xiaoyan C. (2010). Characteristics of environmental factors and their effects on CH 4 and CO 2 emissions from a closed landfill: an ecological case study of Shanghai. Waste Manag..

[27] Xi B.-D., He X.-S., Wei Z.-M., Jiang Y.-H., Li M.-X., Li D. (2012). Effect of inoculation methods on the composting efficiency of municipal solid wastes. Chemosphere.

[28] Sil A., Kumar S., Kumar R. (2014). Formulating LandGem model for estimation of landfill gas under Indian scenario. Int. J. Environ. Technol. Manag..

[29] Alexander A., Burklin C., Singleton A. (2005). Landfill Gas Emissions Model (LandGEM) Version 3.02 User’s Guide.

[30] Eleazer W.E., Odle W.S., Wang Y.-S., Barlaz M.A. (1997). Biodegradability of municipal solid waste components in laboratory-scale landfills. Environ. Sci. Technol..

[31] Staley B.F., Barlaz M.A. (2009). Composition of municipal solid waste in the United States and implications for carbon sequestration and methane yield. J. Environ. Eng..

[32] Tsatsarelis T., Karagiannidis A. (2009). Estimation of future methane production from Hellenic landfills. Glob. Nest J..

